# PK/PD modeling of 5‐hydroxytryptophan (5‐HTP) challenge test with cortisol measurement in serum and saliva

**DOI:** 10.1002/prp2.574

**Published:** 2020-03-13

**Authors:** Zheng Guan, Gabriel Jacobs, Hans van Pelt, Joop M.A. Van Gerven, Jacobus Burggraaf, Wei Zhao

**Affiliations:** ^1^ Centre for Human Drug Research Leiden the Netherlands; ^2^ Leiden University Medical Center Leiden the Netherlands; ^3^ Northwest Clinics Alkmaar the Netherlands; ^4^ Department of Clinical Pharmacy School of Pharmaceutical Sciences Shandong University Jinan China

**Keywords:** 5‐HTP, cortisol, modeling, population pharmacokinetics and pharmacodynamics, saliva sampling

## Abstract

This research was planned to build a Pharmacokinetic/Pharmacodynamic (PK/PD) model of 5‐hydroxytryptophan (5‐HTP) challenge study including a circadian rhythm component of cortisol and to predict serum cortisol based on saliva cortisol. Data from three 5‐HTP challenge studies in healthy volunteers were collected. Serum 5‐HTP, saliva, and serum cortisol were sampled as PK and PD marker. The population PK/PD modeling approach was applied. A baseline model of serum cortisol was built to assess the circadian rhythm before a pharmacodynamic model was used to evaluate the drug effect of the 5‐HTP on cortisol. Finally, linear and power function relationships were tested to predict serum cortisol based on saliva cortisol. The PK of 5‐HTP could be described using a one‐compartment model with a transit compartment. The typical value for clearance was 20.40 L h^−1^ and showed inter‐study variability. A cosine function was chosen and properly described the circadian rhythm of serum cortisol. A linear approximation model was applied to fit the 5‐HTP PD effect on cortisol data with a slope of 4.16 ng mL^−1^ h. A power function provided a better description than a linear function to relate the saliva and serum cortisol. In conclusion, a circadian rhythm component was built in the PK/PD model of the 5‐HTP challenge test which could better improve the understanding of the stimulating effect on HPA with cortisol change. After the 5‐HTP challenge, saliva cortisol correlated well with serum cortisol and was predictable by a population PK‐PD model.

**What is known about this subject?**

We have previously demonstrated reproducible, concentration‐dependent pharmacodynamic effects with acceptable variability associated with a serotonergic function test in healthy volunteers using 5‐hydroxytryptophan to guide the development of the novel compound that target central components of the HPA axis. The evaluation of the circadian rhythm effect of cortisol which is the biomarker of the challenge test and the possibility of using saliva cortisol as an alternative monitor metric will assist our understanding of this challenging test.

**What this study adds？**

This study retrospectively collected the data of three trials of the 5‐HTP challenge test in healthy volunteers. Population PK/PD modeling which chose both serum and saliva cortisol as observations was constructed incorporating the circadian rhythm of cortisol. This improved the understanding of the 5‐HTP stimulating effect on the HPA axis and provided the possibility of applying the salivary sampling of cortisol as a monitor metric due to its less burdensome and better feasibility.

## 1 INTRODUCTION

The hypothalamus‐pituitary‐adrenal axis (HPA) has been associated with the neurobiology of mood and anxiety disorder, etc.^1^ A reliable and well‐characterized pharmacological challenge test that quantitatively evaluates the function of central components of the HPA axis, would, therefore, be a useful tool to evaluate its role health and psychiatric disease. In addition, such challenge study could be helpful delineating endophenotypal characteristics of clinical psychiatric phenomena and guiding the development of innovative central nervous system (CNS) drugs along a rational path.

5‐hydroxytryptophan (5‐HTP) is converted from tryptophan, an essential amino acid, by tryptophan hydroxylase and it is further converted to serotonin. 5‐HTP has been used in alternative medicine as a possibly effective aid in treating depression or fibromyalgia and its potential applications under research include insomnia, alcohol withdrawal, migraine, premenstrual syndrome, binge‐eating related to obesity, attention deficit disorder, cerebellar ataxia, and muscle spasms in the mouth.^2^, ^3^, ^4^, ^5^, ^6^, ^7^ 5‐HTP is also reported as a challenge test to examine central serotonergic function, with cortisol and prolactin release used as a measure of response, as well as the excretion of the metabolite 5‐hydroxy‐indoleacetic acid.^8^, ^9^, ^10^, ^11^, ^12^ In previous studies, we have demonstrated reproducible, concentration‐dependent pharmacodynamic effects with acceptable variability associated with a serotonergic function test in healthy volunteers using 5‐hydroxytryptophan (5‐HTP).^8^, ^9^ Carbidopa and granisetron were co‐administrated with 5‐HTP. Carbidopa prevented the peripheral conversion of 5‐HTP to 5‐hydroxytryptamine (5‐HT) which would preclude brain penetration, while granisetron limited serotonergic side‐effects such as gastro‐intestinal stimulation and vomiting without influencing the neuroendocrine response or 5‐HTP pharmacokinetics.^9^ The 5‐HTP challenge test is used to quantify central serotonergic (5‐HT) neurotransmission by elevating central 5‐HT. The increase of 5‐HT activates the HPA axis which then releases corticotrophin (CRH), adrenocorticotrophic hormone (ACTH), and cortisol step by step. Cortisol was tested as the neuroendocrine endpoint as a key and a downstream steroid hormone of the HPA axis which is involved in stress and different diseases.^13^, ^14^, ^15^

In human plasma, the major fraction (about 70%) of cortisol is bound to corticosteroid‐binding globulin (CBG), approx. 20% is bound to albumin and 10% is unbound.^16^ Several observations have led to the conclusion that only the unbound cortisol is able to penetrate the intracellular compartment and that the CBG‐cortisol complex has no direct hormonal activity.^17^, ^18^ Cortisol measurement in blood samples is easily interfered with stress.^19^ The invasive blood sampling can cause an up‐swing of cortisol serum concentration. The up‐swing artificially generates a high concentration and cannot reflect the true concentration. One way to avoid this artificially high concentration cause by blood draw may be using non‐invasive measurement like salivary sampling. There are already cases and attempts to use saliva to the predict serum/plasma concentration of drugs in many therapeutic and research areas, such as antituberculosis drugs, anticonvulsants, antiepileptic drugs, psychobiological agents, etc.^20^, ^21^, ^22^, ^23^ Salivary sampling is a non‐invasive patient‐friendly method, which offers new possibilities for cortisol measurement since it can also be sampled when volunteers or patients are at home. Salivary cortisol concentration reflects the biologically active serum unbound cortisol level and is thus unaffected by elevations in CBG, which confuse the interpretation of serum cortisol levels.^24^ As a result, another advantage of testing salivary sampling is that the distribution of cortisol from blood to saliva generally occurs by passive diffusion and different researches have shown that the salivary cortisol concentration correlates well with the serum‐free cortisol concentration throughout the physiological concentration range.^9^, ^24^, ^25^, ^26^, ^27^ Under normal physiological condition without a pharmacological challenge, the relationship between serum and saliva cortisol has already been studied with regression analysis.^28^, ^29^ However, after a challenge of 5‐HTP, the higher range of cortisol in both serum and saliva should further be studied.

Another complicating factor of cortisol after the 5‐HTP challenge is the fact that the concentration of cortisol follows circadian rhythm. Plasma concentration of cortisol reaches peak concentrations in the morning (6 AM to 10 AM) and trough concentration at night between 8 PM and 2 AM.^30^, ^31^, ^32^, ^33^ As a result, the change from baseline of cortisol after the 5‐HTP challenge is the mixed additive effect of drug response and circadian rhythm. To better understand the effect of 5‐HTP, a circadian rhythm factor should be peeling off from the total change after baseline.

In the current study, we retrospectively collected data from three studies conducted in our research center within 5 years. The combination of data was based on the fact that all three trials were designed similarly so that the heterogeneity of these trials was small. Data were pooled to enable us to utilize a population pharmacokinetic (PK)/ pharmacodynamic (PD) modeling approach for addressing the aforementioned issues. PK/PD modeling is an approach to characterize the concentration‐time profile and the relationship between concentrations and effects using a mathematical model. Model estimation can be based on both individuals and populations. The assumption that all individual concentration‐effect relationships can be described with the same structural model is based on the notion that the drug activates the same pharmacological system in all subjects (or systems for different responses). PK/PD modeling is performed by a non‐linear mixed effect modeling approach which provides the estimates of the population average parameters (assuming that each individual can be described using the same structural model) and their associated inter‐individual variability, which allows individuals to differ from each other. Residual error describing the variability of the difference between predicted values and the observations is also estimated.^34^, ^35^ In the present study, we constructed a population PK/PD model. PK of 5‐HTP and its effect on cortisol level in serum and saliva with consideration of circadian rhythm could be investigated on both population and individual level. It was also possible to mathematically describe the circadian rhythm phenomenon of cortisol into the model. After identifying the circadian rhythm factor, the working pattern of the 5‐HTP effect on the HPA axis could be better learned in terms of onset time, effect size, etc

In summary, the aims of the current study were as follows: 1) Develop a population PK/PD model for the effect of the 5‐HTP challenge test on acute serum cortisol increases incorporating circadian rhythm component; 2) Explore the relationship between saliva cortisol and serum cortisol using the population approach.

## 2 MATERIALS AND METHODS

### 2.1 Clinical trial design

**FIGURE 1:**
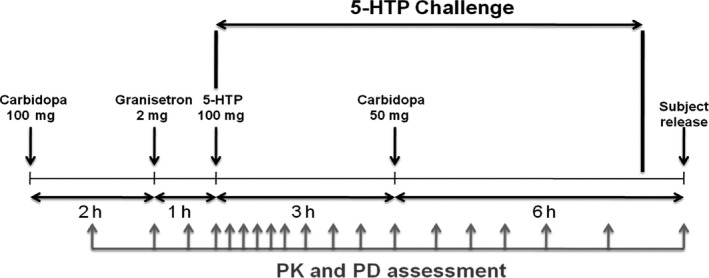
5‐HTP challenge trial design scheme

We retrospectively included three studies (CHDR0204, CHDR0612, and CHDR0716). The three trials were randomized, double‐blind, double‐dummy placebo‐controlled, crossover trial, and were performed at CHDR. The combination of 5‐HTP (200 mg), CBD (100 mg + 50 mg), and granisetron (2 mg) was orally administrated. Carbidopa was administrated to prevent peripheral carboxylation which can stabilize the PK of 5‐HTP and granisetron was administrated as an antiemetic to reduce the systemic side‐effects of 5‐htp.^8^, ^9^ The sampling time started before the administration of 5‐HTP and finished 5 to 9 hours after 5‐HTP administration. The 5‐HTP challenge trial designs scheme were separately described in previous publications and summarized in Figure 1.^8^, ^36^, ^37^

The study protocols were approved by the Medical Ethics Committee of Leiden University Medical Centre and performed according to the Good Clinical Practice and International Conference on Harmonization guidelines. Further utilization of the data in later scientific research from these studies was noticed to all subjects in informed consents which were available per request.

In total, 35 healthy male volunteers participated in these studies. Their blood and saliva samples were collected. The plasma of 5‐HTP, total serum cortisol, and saliva cortisol concentrations were measured. Study medications and biochemical methodologies of PK and PD measurements can be found in previous publications as well.^8^, ^12^, ^36^, ^37^ The demographic data of subjects were also collected.

Saliva was collected using cotton wool swabs (Salivette, neutral; Sarstedt Rommelsdorf, Germany) in which subjects placed in their mouth and chewed for approximately 45‐60 seconds. Saliva cortisol concentrations were measured with a time resolved fluorescence immunoassay on a Hitachi apparatus (Roche) at the central laboratory for clinical chemistry (CKCL) of Leiden University Medical Centre, Leiden.

Additionally, in two studies, subjects’ corticosteroid‐binding protein (CBG) concentrations were measured. Then free serum cortisol could be calculated later using the method of Coolens et al^26^ The Coolens equation is based on the total serum cortisol and CBG concentrations, considering the affinity of cortisol for CBG and albumin as below:

(1) $$U=\sqrt{Z^{2}+0.0122T}-Z$$

(2) $$Z=0.0167+0.182\left(G-T\right)$$

where *U* is the free serum cortisol concentration, *G* is the CBG, and *T* is the total serum cortisol. CBG was analyzed using a radioimmunoassay kit from the BioSource (Nivelles, Belgium) at the Xendo Drug Development BV, Groningen, The Netherlands.

### 2.2 Population approach

The population approach using nonlinear mixed‐effects models was performed using NONMEM 7.1.0. The method used was the First‐order conditional estimation (FOCE). Parameters were estimated with possible inter‐individual variability (IIV) in the followed statistical model. IIV was exponentially expressed using Equation 3:

(3) $$P_{\mathrm{ij}}=P_{TV_{j}}\cdot \exp\left(\eta_{\mathrm{ij}}\right)$$

In the above equation, *P*_ij_ represented the *j*_th_ basic pharmacokinetic parameter of the *i*_th_ individual. All the values of *P*_ij_ were assumed to be log‐normally distributed. $P_{{\text{TV}}_{j}}$ was the typical population value of the *j*_th_ parameter and *η_ij_* is the deviation of *P*_ij_ from $P_{{\text{TV}}_{j}}$ with a mean of 0, and an estimated variance of $\omega_{j}^{2}$.

Both proportional and additive error model were tested to describe the residual unexplained variance between the observed concentrations and predictions from the model. The combined residual error model which combines proportional and additive error structures was also tested. The residual error statistical model followed Equation 4.

(4) $$O_{\text{obs}}=O_{\text{pred}}\cdot \;\left(1+\varepsilon_{1}\right)+\varepsilon_{2}$$

*O*_obs_ and *O*_pred_ represented the observed and predicted Observations including both PK and PD model, respectively. *ε*_1_ and *ε*_2_ represented random deviation between the predicted and observed concentration, with a zero mean and variances of $\sigma_{1}^{2}$ and $\sigma_{2}^{2}$.

### 2.3 PK/PD modeling

The population approach was applied to analyze both PK and PD data. The compartmental model was used to fit the pharmacokinetic profile of 5‐HTP during the challenge test. One‐, two‐, and three‐compartment models and different elimination kinetics were used to fit the pharmacokinetic profile of 5‐HTP in plasma. Different approaches for absorption phase fitting were tested including transit compartment and the use of lag time. Model development was guided by comparing an objective function value (OFV) based on the −2 × log likelihood (−2LL) of increasingly more complex models and standard goodness of fit plots.

A two‐step modeling approach was used. First, the PK model for 5‐HTP was built to obtain the estimated PK parameters based on OFV and goodness of fit. The PK model was only built to optimally describe the PK profile. Second, the PK/PD model was built. Individual empirical Bayes’ estimates were determined to describe the concentration profile and used in the subsequent PK/PD analyses.

To build the PD model of stimulated serum cortisol concentration, a baseline model of serum cortisol was first built to assess the circadian rhythm based on the data of the placebo group using a cosine function as Equation 5.^38^

(5) $$\text{BSL}={\text{BSL}}_{0}\times \left(1+\text{AMP}\times \text{Cos}\left(\frac{2\pi \times \left(\text{Time}-\text{Tpeak}\right)}{n}\right)\right)$$

BSL_0_ was an initial baseline value. AMP was the amplitude of the cosine term. n could be different values (such as 4, 8, 12, 16, 24, etc) and the final chosen value should be suggested by the model fitting procedure. BSL was the total apparent baseline.

Then, a sigmoid model was selected to model the drug effect of the 5‐HTP challenge as Equation 6. There was no reported evidence that the circadian rhythm of cortisol is affected by stress or drugs. As a result, total plasma cortisol (as E in Equation 6) was calculated as the sum of the effect of 5‐HTP challenge part and baseline level of cortisol.^38^, ^39^, ^40^, ^41^

(6) $$E=\text{BSL}+\frac{E_{\max}\cdot C_{5-\text{HTP}}}{EC_{50}+C_{5-\text{HTP}}}$$

where *E*_max_ is the maximum stimulation effect of 5‐HTP and EC50 is the 5‐HTP concentration producing 50% of maximum stimulation. In the modeling process, a shift in the circadian rhythm was discovered visibly between‐day variability. Accordingly, inter‐occasion variability was included to describe the day to day differences of the individual baselines.

Linear and power functional relationships were used to predict the saliva cortisol based on serum cortisol which was presented by total serum cortisol or free serum cortisol separately.

(7) $$C_{\text{sal}}=\beta \times C_{\text{col}}^{\gamma }$$

*β* serves as a simple scaling factor. *γ* is called either the exponent or the power, which determines the function's rates of growth or decay and the function's overall shape and behavior. If *γ* equals 1, the relationship becomes linear. *C*_sal_ represents saliva cortisol concentration. *C*_col_ represents either serum total or serum‐free cortisol concentration. The final choice of using which one in the final model will be determined by the model fitting result.

Visual predictive checks (VPC) were performed for all PK and PD models using R version 2.12.0 (R: A Language and Environment for Statistical Computing, R Development Core Team, R Foundation for Statistical Computing, Vienna, Austria, 2010) with the lsoda (deSolve Package 1.8.1) and mvrnorm functions (MASS Package v7.3‐8). The visual predictive check encompassed a projection of the simulated‐dependent variable as a function of time using the final model on the observations. The simulations were performed considering the estimated population parameters (Θ vector) as well as the covariance matrix describing IIV (Ω matrix). The residual variability (Σ matrix) was not included in the simulations. The simulations and data were grouped by the antagonists’ dose. Summary statistics of the simulations (median and the 95% prediction interval of the simulated IIV) enabled a comparison of the predicted and the observed variability. For each dose group, 1000 individuals were simulated.

### 2.4 Software

NONMEM version 7.1.0 (Beal, S., Sheiner, LB, Boeckmann, A., & Bauer, RJ, NONMEM User's Guides. (1989‐2009), Icon Development Solutions, Ellicott City, MD, USA, 2009)^34^ was used for nonlinear mixed effect modeling and R version 2.12.0 (R Development Core Team, R Foundation for Statistical Computing, Vienna, Austria, 2010) was used for data file preparation, diagnostic plotting, simulation, and visual predictive check.

## 3 RESULTS

### 3.1 Subject information

We included a total of 35 healthy male volunteers. The numbers of each type of observation in three trials are shown in Table 1. For all of the subjects, the mean age was 25.25 ± 7.24 years; mean height was 1.83 ± 0.056 m; average weight was 78.02 ± 9.49 kg; and the average CBG was 45.94 ± 5.94 mg L^−1^. The disposition of the volunteers has been published separately before.^8^, ^36^, ^37^

**TABLE 1:** Brief information of observations

Project	Sub. No.	Observation (5‐HTP)	Observation (serum cortisol)	Observation (salivary cortisol)
CHDR0204	13	138	263	0
CHDR0612	11	77	341	169
CHDR0712	11	64	204	87
Total	35	279	808	256

### 3.2 PK of 5‐HTP in challenge test

**FIGURE 2:**
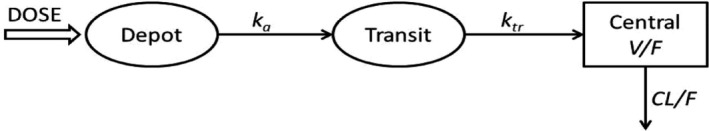
Schematic illustration of the population pharmacokinetic model of oral administered 5‐HTP. CL, clearance; F, oral bioavailability; ka, oral absorption rate; ktr, transit rate constant; V, volume of distribution. ka = ktr

A one‐compartment model with first‐order absorption and elimination was used to describe the pharmacokinetics of 5‐HTP during the challenge test. A transit absorption compartment dominated the “time lag” and was introduced to improve the description of the concentration upswing after oral drug administration. The schematic illustration of the population pharmacokinetic model is shown in Figure 2.

**FIGURE 3:**
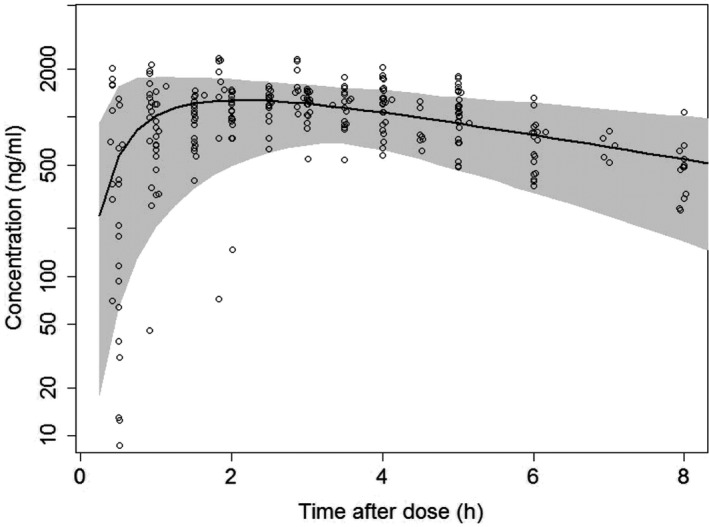
Visual predictive check of 5‐HTP concentration‐time profiles. Open circles represent observations, line and gray areas represent predicted mean and 95% confidence interval, respectively

The PK parameters are presented with parameter estimate, relative standard error, and inter‐individual variability in Table 2 (also refer to Supplement material S1). Inter‐individual variability (IIV) was identified on the apparent clearance (CL/F) and absorption rate (Ka). Only proportional residual error was included in the model. VPC showed that most of the data fell within the 95% prediction interval and were symmetrically distributed around the median (Figure 3), which suggested that the final model adequately described the majority of the data. Diagnostic plots also supported that the model fitted 5‐HTP PK data properly (Figure S1). In diagnostic plots, different colors were marked for three studies, from which it was shown that the final model fitted observations equally acceptable in all studies.

**TABLE 2:** Population model parameters with relative standard error and inter‐individual variability

Model	Parameters	Estimates	RSE(%)	ω^2^	IOV
5‐HTP PK model	CL/F (L/h)	20.40	7.64	0. 16	—
	ka (h^−1^)	1.89	12.10	0.38	—
	V/F (L)	102.00	5.32	—	—
	*σ* (ng/mL)	0.11	14.10	—	—
Cortisol circadian rhythm model and PD model	*S*_0_	0. 072	11.40	0. 31	—
	Baseline(ng/mL)	88.60	5.09	0. 056	0. 049
	Amplitude	−0. 23	−12.30	0. 086	—
	Trend(ng/mL.h)	4.16	5.69	0. 17	—
	*T*_peak_(h)	11.50	—	0. 021	—
	*σ* (ng/mL)	0. 069	10.90	—	—
Saliva cortisol model	*γ*	1.10	8. 27	—	—
	*β*	1.01	36.00	0.081	—
	*σ* (nmol/L)	0.23	11.80	—	—

RSE, Relative standard error = standard error/estimate; ω^2^, inter‐individual variability; σ is the residual error; Ka, absorption rate constant; V/F, apparent distribution rate; CL/F, apparent clearance.

### 3.3 Circadian rhythm for total serum cortisol baseline and pharmacodynamic model

A cosine function^42^, ^43^ was used to describe the circadian rhythm of serum cortisol according to the observed sampling day time. As in Equation 5, different choices of n (4, 8, 12, 16 and 24) were test to identify the best value to describe the circadian rhythm and 8 was finally chosen. Also, a trend part was added to better fit the shape of a gradually decreasing of serum cortisol at each day based on an OFV drop of 76.33 and better performances of diagnostic plots (see Equation 8 where Trend was the slope of both linear decreasing and cosine fluctuation). Within this function, four parameters were included in Equation 8. Both IIV and inter‐occasion variability ((ICV)) were identified and included in the final model. IIVs were added to all baseline model parameters and ICV was found necessary to BSL_0_. The parameters were presented with parameter estimates, relative standard error, IIV and ICV in Table 2 (also refer to Supplement material *S2*).

(8) $$\text{BSL}=\left({\text{BSL}}_{0}-\text{Trend}\times \left(\text{Time}-\text{Tpeak}\right)\right)\times \left(1+\text{AMP}\times \text{Cos}\left(\frac{2\pi \times \left(\text{Time}-\text{Tpeak}\right)}{8}\right)\right)$$

The challenge test involved only one dose level of 5‐HTP, which somehow prevented estimating both the *E*_max_ and *EC*_50_ in the sigmoid model. Instead, an approximation with the linear model was applied in the absence of a plateau effect of the drug. When C_5‐HPT_ was much less than EC_50_, the *E*_max_ model was approximated to a linear model with the intercept of BSL and slope of *E*_max_/EC_50_, which was named *S*_0_. Only proportional residue error was included. As a result, the final model of the PD part changed to Equation 9 as below:

(9) $$E=\text{BSL}+S_{0}\cdot C_{5-\text{HTP}}$$

**FIGURE 4:**
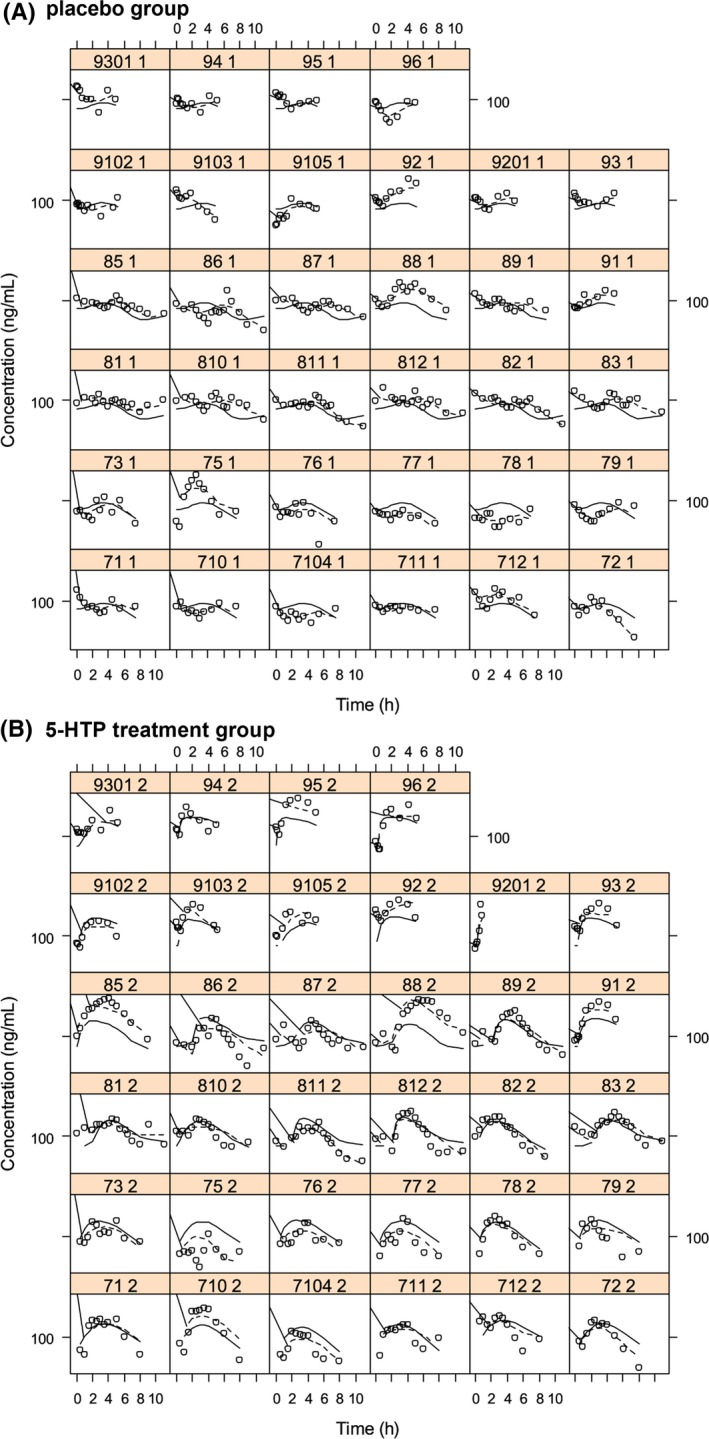
Trellis plot for total serum cortisol. Open circles: observations, solid line: population modeling prediction, and dashed line: individual modeling prediction. The lables in the head of each grid are the subject identification informations including trial number, subject code and treatment code

The RSE showed the acceptable accuracy of the parameter estimate and no obvious shrinkage was found. Different trellis plots were drawn to test the model propertied. In Figure 4, for each subject, observation, population prediction and individual prediction were plot in the same panel, which showed a proper prediction of the individual line and an obvious variation between subjects. The model predicted both the placebo group and treatment group properly. Diagnostic plot and individual trellis plots were drawn to validate that the final model worked properly to fit observed data. VPC could not be performed for this model since the time variables for each subject involved two types which were time after dose and day time. The different combinations of these two time variables for each subject resulted in the infeasibility of running a VPC. Alternatively, individual trellis plot prediction and diagnostic plots were chosen to be the final validation approach of the model (Figure 4 and Figure S2). In diagnostic plots, different colors were marked for three studies, from which it is shown that the final model fit observations equally acceptable in all studies.

### 3.4 Relationship between serum and saliva cortisol

**FIGURE 5:**
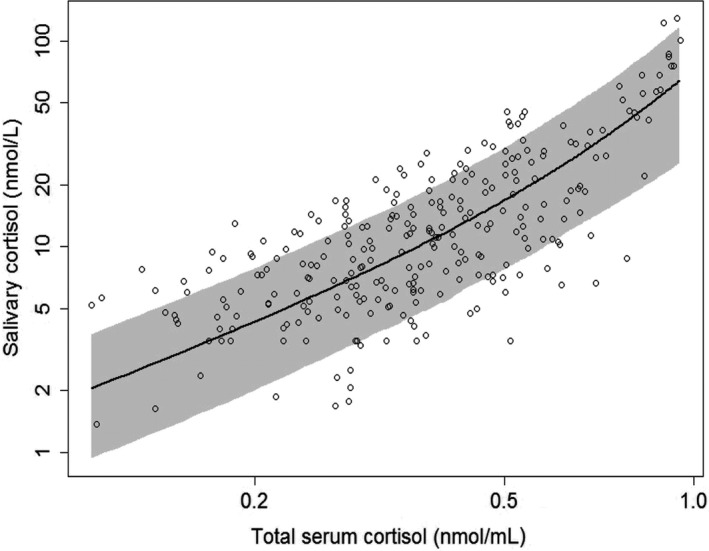
Visual predictive check of salivary cortisol versus total serum cortisol concentration relationship. Open circles represent observations, line and grey areas represent predicted mean and 95% confidence interval, respectively

A power function (Equation 7) provided a better description than a linear function to relate saliva cortisol with serum cortisol. Additionally, free serum cortisol was a better predictor for saliva cortisol than total serum cortisol. The parameters are presented with parameter estimate, relative standard error, and inter‐individual variability in Table 2 (also refer to Supplement material *S3*). A VPC was performed to verify the model performance which showed that most of the data fell within the 95% prediction interval and were symmetrically distributed around the median. The VPC and diagnostic plots are shown in Figure 5 and Figure S3. In diagnostic plots, different colors were marked for different studies, from which it is shown that the final model fit observations equally acceptable in all studies. Only CHDR0607 and CHDR0712 included saliva cortisol data so that only data of these two trials were used to build the correlation model of cortisol in saliva and serum.

## 4 DISCUSSION

Our aims were to develop a population PK/PD model for the effect of the 5‐HTP challenge test on acute serum cortisol increases incorporating circadian rhythm component and to explore the relationship between saliva cortisol and serum cortisol using the population approach.

The presented model was the first population PK model developed for 5‐HTP after the co‐administration of 5‐HTP, carbidopa, and granisetron. The result was consistent with previous publications with respect to parameter estimates and a high inter‐subject variability.^9^ The absorption and elimination half‐life estimates were 0.367 and 3.47 hours, respectively. Despite the considerable inter‐individual variability (IIV), the model accurately predicted the serum 5‐HTP concentration on an individual level. The side effect of 5‐HTP challenge test was found to be related to PK exposures.^9^ The current model provided a PK model that can predict PK exposure in population and individual levels, so that it may serve as a tool to predict or explain the occurrence of side effects in later 5‐HTP challenge studies.

Only one 5‐HTP dosage level (200 mg) was available for modeling in the present study. Since 200 mg was the selected dosage level of 5‐HTP challenge based on a series of previous studies and was used as a benchmark in the same type of pharmacological challenge trial in the future,^8^, ^9^, ^36^, ^37^ 200 mg represented the most clinical relevant dose. Besides, earlier publications demonstrated the linearity of 5‐HTP PK within a range of oral administration from 100 to 300 mg.^9^ Yet, the application of the current model to extrapolate for higher dosage of 5‐HTP should be cautious.

In this work, the presented cosine function with a trend served as a model to capture the daily profile of cortisol in a descriptive manner. All the parameters used in the function were found to be necessary with an IIV. However, from individual aspects, based on the placebo occasion result, the model delineated the observed circadian rhythm well. It was understandable that with the assistance of baseline information from the placebo group, the effect of 5‐HTP in the treatment group was better predicted with an individual baseline deducted even though a shift of the baseline might exist between the two occasion days within the same subject. The better identification of the challenge effect should be attributed to the use of population modeling and this approach can reduce bias comparing with a previous statistical description of the drug effect without deducting baseline noise from circadian rhythm.

Moreover, in the present studies, the sampling time period was still not enough to delineate the whole daily time course. In the presented model, the cosine function part was used to mimic the curling shape and cycling property of the circadian rhythm, while the trend with a negative slope was meant to simulate a general decreasing tendency within the observed time period which was chiefly from 11 AM to 8 PM. If the model was applied and extrapolated incautiously to a later time in the night time, the use of trend would produce bias of underestimation of the cortisol level. Longer sampling, including the night time and early time in the morning, could offer a chance to depict a better picture of the circadian rhythm of serum cortisol.

A linear relationship was built between serum 5‐HTP concentration and total serum cortisol. The direct effect between drug concentration and serum cortisol was found good enough to build the PK/PD relationship. No obvious hysteresis was found during the steps of mode fitting. This suggested that even though 5‐HTP stimulated the creation of cortisol through the whole HPA axis, the steps in between could be treated as a very fast process and then these consequent steps could not be reflected in the modeling component. However, if different mechanisms, such as feedback process, are also under consideration, a more complicated and mechanism‐based model with different steps including ACTH or CRH may also be included in the model. Yet, in the presented model, the linear direct effect relationship predicted individual well with proper parameter estimates and RSEs, which is a robust starter to build the relationship between serum cortisol and saliva cortisol after the 5‐HTP challenge.

As a similar situation of PK, only one dosage level of the 5‐HTP administration was tested in the model which limited the possibility of obtaining a wider exposure‐response relationship between 5‐HTP and serum cortisol. As a result, when building the PD model, the sigmoidal model could not be constructed but the linear relationship. About 200mg of 5‐HTP was selected and will be used as a routine challenge dosage in the future, which made this limitation less important. However, when a higher dosage of 5‐HTP is given with other purposes, the PK/PD relationship presented here may need adaptation.

A power function model was selected to build the relationship between serum and saliva cortisol in the population approach instead of the published regression approach.^28^, ^29^ This provided not just population estimates but also individual prediction. By simultaneously fitting the PK‐PD model and power function model, the result showed a predictive ability from 5‐HTP administration to saliva cortisol according to the model validating methods. From the literature, both free serum cortisol and total serum cortisol were used to build the regression model with saliva cortisol and free serum cortisol showed better correlation^24^, ^28^ as the free part represents the available part of cortisol which can freely diffuse from blood and saliva. Our study result supported the observations of previous publications.^24^, ^28^ The wide inter‐individual variability in the presented model was observed. On the one hand, this variability naturally existed due to the complicated physiological process. Salivary pH value, salivary flow rate, and pathological event of the oral cavity were all factors that could have an impact on the individual cortisol salivary concentration. One the other hand, by collecting these physiological variables if feasible in future studies, the current wide inter‐individual variability in the model can be decreased and part of the variability could be included and better explained in the structure model. Besides, standardized and well‐controlled sampling conditions should be strictly observed and precautions have to be taken to avoid potential impacts on study outcome. In our presented study, free cortisol was not directly measured but calculated based on the Coolens’ equation with measured CBG concentration. Our research only applied the Coolens’ conclusion but did not validate it. A study with simultaneous measurement of free serum cortisol as well might give a better clue of the validation of Coolens’ equation.

The range of normal daily range of total serum cortisol is from 140httpcite_note-goodhope-15://en.wikipedia.org/wiki/Cortisol ‐ cite_note‐goodhope‐15 to 700 nmol L^−1^ in the day time and 80 to 350 nmol L^−1^ in night time.^42^ After the 5‐HTP challenge, it increased to 1000 nmol/L. The used power function in the model suggested a nonlinear relationship between free serum cortisol and saliva cortisol which was especially observed in high concentration range after 5‐HTP challenge, but the predictive quality kept almost the same within the whole range which could be seen from the VPC plot. It was fair to conclude that the fast stimulus from 5‐HTP to the HPA axis does not influence the fast diffusion of cortisol between serum and saliva so that the prediction of saliva cortisol based on free serum cortisol was feasible. In the clinical trial, using saliva sampling as an alternative way of blood drawing would benefit from the compliance aspect and be with reasonable predictive capability without interference from stress.

One of the limitations of this research was that only male subjects were included. The cortisol level and change in female subjects were reported to be different. While some studies reported higher baseline cortisol levels in men,^44^, ^45^, ^46^, ^47^, ^48^ no differences were found in other investigations.^49^, ^50^, ^51^, ^52^, ^53^ It should be noted that lower concentrations were found only in females during the follicular phase. Cortisol levels were comparable with men when measured in the luteal phase.^44^, ^45^, ^46^, ^47^ Similarly, cortisol responses to stimulation yielded heterogenous results. Cortisol responses to stimulation yielded heterogenous results. Larger increases of cortisol in men were observed following 5‐hydroxytryptophan administration^54^ and no sex differences in cortisol responses were observed under physical stress.^51^, ^52^ Similarly, no sex differences in adrenocortical activity could be observed in studies exposing healthy subjects to mild psychosocial stress.^55^, ^56^, ^57^, ^58^ There was also a study reporting difference in the saliva cortisol level between male and female subjects.^59^ These potential gender differences mentioned above may lead to difficulty in directly applying the current PK/PD model in the female subject. Further study with female should be recruited and studied.

In conclusion, the PK/PD model, including a cosine function with a trend served as a simplified model to describe part of the circadian rhythm, could describe and predict the total serum cortisol concentration for the proposed dose level in the 5‐HTP challenge test, but limitations existed when extrapolating to higher dose levels. The relationship between saliva cortisol and serum cortisol was well characterized by a power function. The results provide a rationale to sample cortisol from saliva as an alternative of serum.

## ETHIC STATEMENT

The study was approved by the Medical Ethics Committee of Leiden University Medical Centre and performed according to the Good Clinical Practice and International Conference on Harmonization guidelines. Further utilization of the data in later scientific research from these studies was noticed to all subjects in informed consents that were available per request.

## CONFLICT OF INTERESTS

All authors have completed the Unified Competing Interest form at http://www.icmje.org/coi_disclosure.pdf (available on request from the corresponding author) and declare no support from any organization for the submitted work, no financial relationships with any organizations that might have an interest in the submitted work in the previous 3 years and no other relationships or activities that could appear to have influenced the submitted work.

## Supporting information

Fig S1‐S3

## Data Availability

The data that support the findings of this study are available from the corresponding author upon reasonable request.
