# Supplementary material for: PK/PD modeling of 5‐hydroxytryptophan (5‐HTP) challenge test with cortisol measurement in serum and saliva
Source: Pharmacol Res Perspect. 2020 Mar 13;8(2):e00574. doi: 10.1002/prp2.574 (PMC7069653; doi:10.1002/prp2.574)
Supplement: Supplementary file 1 — Fig S1‐S3 [file PRP2-8-e00574-s001.docx]

**Supplement figure 1-3**

Abbreviations: DV: dependent value; PRED: population predictive value; IPRED: individual predictive value; CWRESI: first order conditional residuals with full interaction


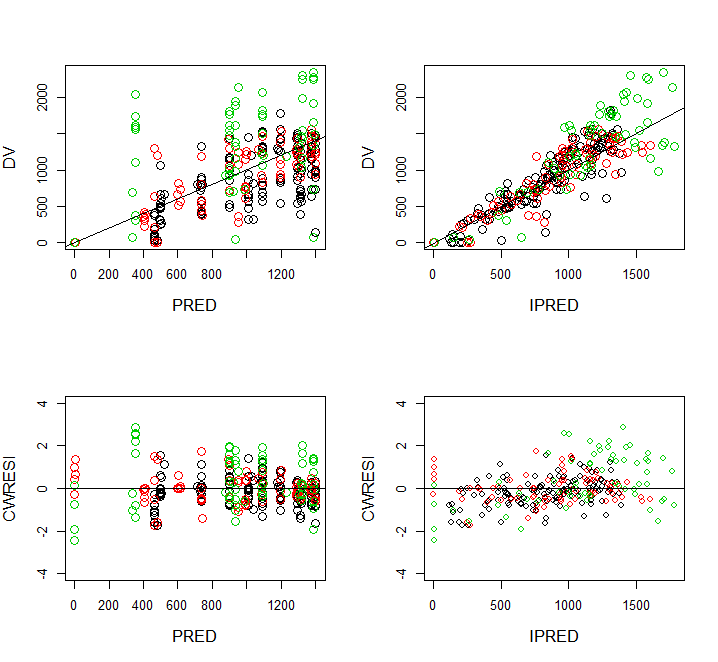


Figure1: Diagnostic plot for 5-HTP PK model: black represents study CHDR0204; red represents study CHDR0612 and green represents study CHDR0716.


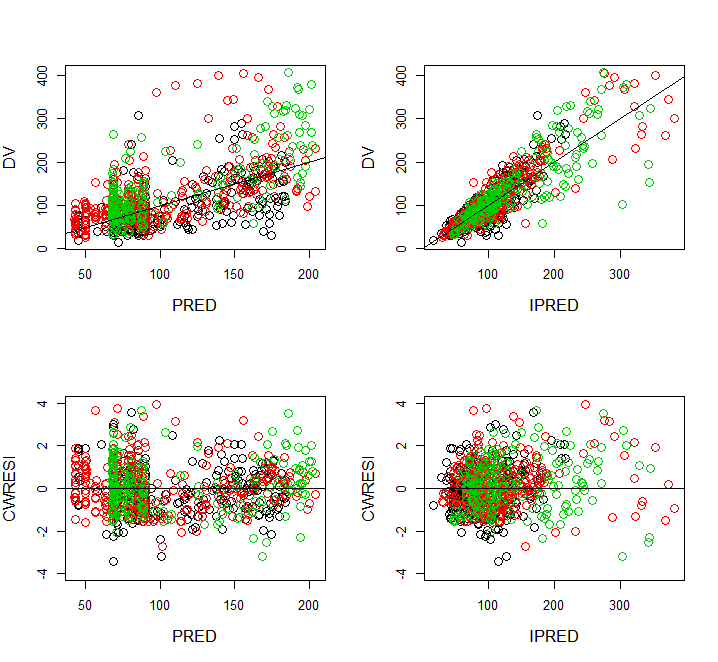


Figure2: Diagnostic plot for cortisol circadian rhythm and PD model: black represents study CHDR0204; red represents study CHDR0612 and green represents study CHDR0716.


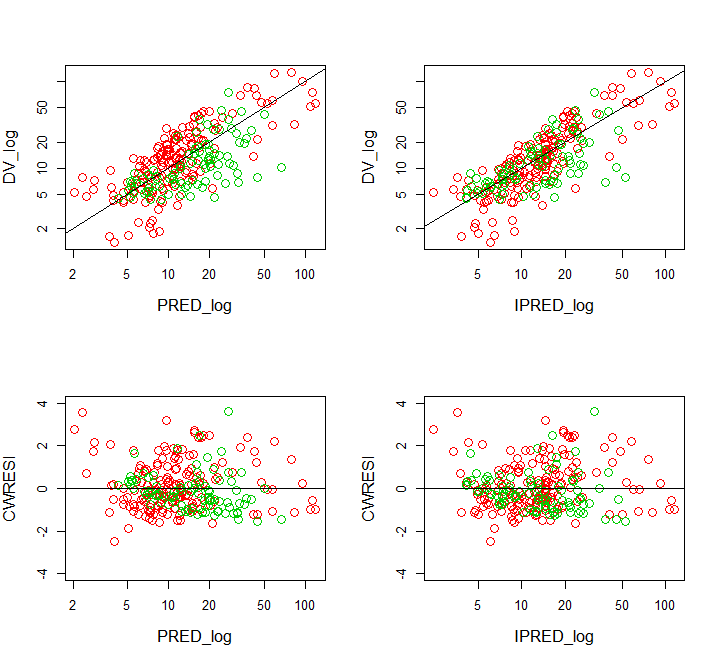


Figure3: Diagnostic plot for relation between serum total and saliva cortisol model: red represents study CHDR0612 and green represents study CHDR0716. For visualizing purpose, the X scales of all four plots and the Y scales of two upper plots are log transformed.
